# Decoding the Distribution of Glycan Receptors for Human-Adapted Influenza A Viruses in Ferret Respiratory Tract

**DOI:** 10.1371/journal.pone.0027517

**Published:** 2012-02-16

**Authors:** Akila Jayaraman, Aarthi Chandrasekaran, Karthik Viswanathan, Rahul Raman, James G. Fox, Ram Sasisekharan

**Affiliations:** 1 Harvard-Massachusetts Institute of Technology Division of Health Sciences and Technology, Department of Biological Engineering, Singapore-Massachusetts Institute of Technology Alliance for Research and Technology, Koch Institute for Integrative Cancer Research, Cambridge, Massachusetts, United States of America; 2 Division of Comparative Medicine, Massachusetts Institute of Technology, Cambridge, Massachusetts, United State of America; University of California San Francisco, United States of America

## Abstract

Ferrets are widely used as animal models for studying influenza A viral pathogenesis and transmissibility. Human-adapted influenza A viruses primarily target the upper respiratory tract in humans (infection of the lower respiratory tract is observed less frequently), while in ferrets, upon intranasal inoculation both upper and lower respiratory tract are targeted. Viral tropism is governed by distribution of complex sialylated glycan receptors in various cells/tissues of the host that are specifically recognized by influenza A virus hemagglutinin (HA), a glycoprotein on viral surface. It is generally known that upper respiratory tract of humans and ferrets predominantly express α2→6 sialylated glycan receptors. However much less is known about the fine structure of these glycan receptors and their distribution in different regions of the ferret respiratory tract. In this study, we characterize distribution of glycan receptors going beyond terminal sialic acid linkage in the cranial and caudal regions of the ferret trachea (upper respiratory tract) and lung hilar region (lower respiratory tract) by multiplexing use of various plant lectins and human-adapted HAs to stain these tissue sections. Our findings show that the sialylated glycan receptors recognized by human-adapted HAs are predominantly distributed in submucosal gland of lung hilar region as a part of O-linked glycans. Our study has implications in understanding influenza A viral pathogenesis in ferrets and also in employing ferrets as animal models for developing therapeutic strategies against influenza.

## Introduction

An important determinant of influenza A virus pathogenesis is the tropism of virus in terms of the specific tissues and cell types that it infects in different host species. The host tissue or cell tropism of the viruses have been investigated previously using *in vitro* pattern of viral adherence (PVA) to tissues from humans and other model animal systems or by staining of fixed *ex vivo* tissue sections with viruses [1], [2]. These studies demonstrated that human-adapted influenza viruses (which show highly efficient respiratory droplet transmission and high pathogenicity in humans) such as H1N1 and H3N2 subtypes specifically bind to human upper respiratory tissues (tracheal/bronchial), whereas avian-adapted viruses (which circulate among birds and have gross pathogenicity only in birds) such as H5N1 bind to human deep-lung and gastrointestinal tract and avian respiratory tissues [1], [2].

Ferrets are widely used as an animal model for understanding influenza A viral pathogenesis and transmission [3]. Upon intranasal inoculation of influenza A virus, ferrets exhibit clinical signs, pathogenesis, and immunity similar to humans upon influenza A infection [4]. Furthermore, ferrets show respiratory droplet transmission of viruses that have adapted to human host but not avian-adapted viruses [5], [6]. However, the tissue and cellular tropism of human-adapted viruses is different between ferrets and humans. In humans, viral binding and infection is observed predominantly in the upper respiratory tract [7], whereas in ferrets, it is observed in lower respiratory tract (specifically in the hilar regions and not as much in the alveolar regions) [8], [9], [10].

One of the important factors governing the tissue or cellular tropism of the virus is the specific binding of its surface glycoprotein hemagglutinin (or HA) to sialylated glycan receptors (complex glycans terminated by sialic acid) on the host cell surface [11], [12]. The sialylated glycan receptors preferentially recognized by human-adapted influenza A viruses (referred to as human receptors) are terminated by sialic acid that is α2→6-linked to the penultimate sugar in the context of at least a trisaccharide motif (Neu5Acα2→6Galβ1-4GlcNAc-) [12]. On the other hand, avian-adapted viruses bind preferentially to α2→3 sialylated glycan receptors (or avian receptors) wherein the terminal sialic acid is α2→3-linked to the penultimate galactose (Neu5Acα2→3Gal) [13], [14], [15]. The distribution of sialylated glycan receptors in cells and tissues of different species has been investigated by histological staining with plant lectins such as *Sambucus nigra* agglutinin (SNA-I) and *Maackia amurensis* lectin (MAL-II) [16]. The glycan-binding specificity of these plant lectins were defined traditionally based on their binding to a specific terminal sialic acid linkage, for example, SNA bound to α2→6- sialylated glycans and MAL bound to α2→3-sialylated glycans.

The advancements in the chemical and chemoenzymatic synthesis of glycans have led to development of microarray platforms comprising of hundreds of diverse glycan structures and structural motifs displayed on various surfaces [17], [18]. Screening the binding of plant lectins and HA on these glycan microarray platforms have permitted the elaboration of their glycan binding specificities going beyond the terminal sugars such as sialic acid linkage [18]. This expanded knowledge on glycan-binding specificities of the lectins and HAs make them valuable tools to obtain more detailed information on glycan structural motifs distributed on different tissue sections.

**Figure 1:**
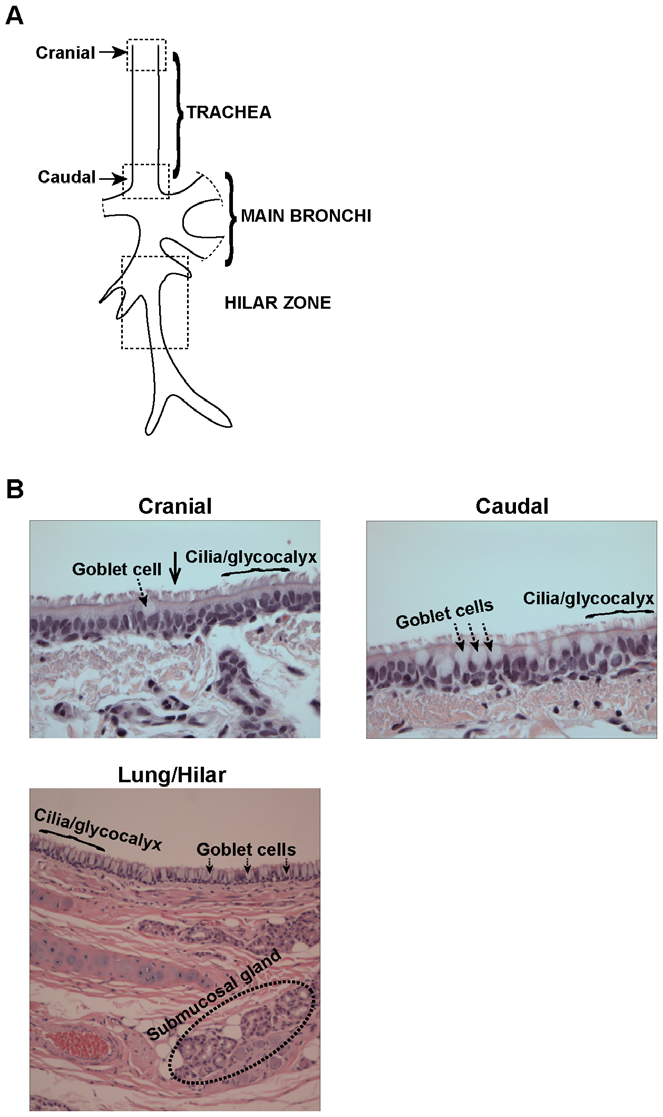
Overview of ferret respiratory tract. (**A**) Regions of ferret respiratory tract analyzed in this study. (**B**) Images of H&E stained ferret tracheal and lung hilar region. As seen from the images, the lung hilar region seems to have more goblet cells than the ferret trachea, a feature distinct from human respiratory tract.

Given the importance of ferrets as widely used animal models for influenza infection, in this study, we have systematically characterized the glycan structural motifs distributed in the tracheal and hilar regions (primary sites of influenza A virus infection) of the ferret respiratory tract (**Figure 1A**). Our study has important implications in better understanding the tropism of influenza A viruses in the established ferret animal model that in turn influences viral pathogenesis.

## Results

### Strategy for the choice of lectins for staining and co-staining ferret tissue sections

SNA-I is a lectin that specifically binds to a terminal disaccharide motif Neu5Acα2→6Gal (or GalNAc)- on any N-linked or O-linked glycan, thereby displaying the broadest specificity to α2→6 sialylated glycans. Therefore staining pattern of SNA broadly indicates distribution of α2→6 sialylated glycans. In order to understand more specifically the distribution of receptors for human-adapted influenza A viruses, recombinantly expressed HAs of two prototypic human-adapted pandemic virus strains from the 1918 H1N1 pandemic, A/South Carolina/1/1918 (or SC18), and from 1957–58 H2N2 pandemic, A/Albany/6/58 (or Alb58) were used. While SC18 has been shown to specifically bind to at least tetrasaccharide glycan motifs terminated by α2→6-linked sialic acid, Alb58 shows a broader specificity by recognizing a minimum trisaccharide motif (Neu5Acα2→6Galβ1-4GlcNAcβ1→). MAL-II was used to characterize distribution of avian receptors. As observed earlier, human-adapted viruses showed predominant binding to non-ciliated (including goblet cells) cells in the upper respiratory tract [19]. Goblet cells are known to predominantly express O-linked glycans as a part of mucins [20], [21]. Jacalin, a lectin with high specificity to Tn antigen (GalNAc-O-Ser/Thr) predominantly found as part of O-linked glycans in mucins, was used to delineate the goblet cell regions in the tissue section. The glycan-binding specificity of the plant lectins and HAs is summarized in **Table S1**.

The lectins and recombinant HAs were used individually or in a multiplexed fashion (co-staining) to delineate glycan structures in regions of the ferret respiratory tract including cranial and caudal parts of the tracheal tissue section and lung hilar section. These regions are known to be infected by human-adapted influenza A viruses. Based on the staining patterns, a detailed picture of the distribution of sialylated glycan structural motifs was obtained that goes beyond terminal sialic acid linkage. The extent of lectin staining of various cell types in the different tissue sections was visually scored and is summarized in **Table 1**. Further, the glycan distribution in ferret respiratory tract was compared to the distribution observed in the human upper respiratory tract.

**Table 1:** Visual scoring of lectin staining of various cell types in tissue sections used in this study.

Lectins used	Cranial			Caudal			Lung Hilar		
	*Glycocalyx*	*Goblet Cells*	*Submucosal gland*	*Glycocalyx*	*Goblet Cells*	*Submucosal gland*	*Glycocalyx*	*Goblet Cells*	*Submucosal gland*
SNA-I	++	+/−	++	++	−	++	+	+	++
MAL-II	−	−	++	−	+/−	++	−	−	−
Jacalin	+	++	++	++	++	++	−	++	++
SNA-I/Jacalin co-staining	−	+/−	++	−	+/−	++	−	+	++
A/Albany/6/58 (Alb58) HA	++	−	++	++	−	++	+	+	++
A/South Carolina/1/18 (SC18) HA	−	−	++	−	−	++	−	−	++
Alb58 HA/Jacalin co-staining	−	+	++	−	+	++	−	+	++

− No staining + Moderate staining +/− Only few cells stained (<10%) ++ Extensive staining.

### Hematoxylin and Eosin staining of Ferret Respiratory Tract tissue sections

Paraffinized tissue sections (5 µm thickness) were stained with hematoxylin and eosin to identify distinct cell types and compare it with that of human respiratory tract. Tracheal tissue sections from the cranial and caudal regions and lung hilar region were analyzed (**Figure 1B**). Both ciliated and non-ciliated goblet cells (which store mucins that are heavily O-glycosylated) were identified in all the three regions. The relevance of understanding distribution of goblet cells arises from previous studies that have shown that HA from human-adapted viruses show substantial binding to these cells in human tracheal section [22], [23] and human-adapted viruses have also been shown to predominantly infect these non-ciliated cells [24]. In the H&E stained sections, the goblet cells were identified as unstained “goblet-shaped” cells in the epithelium (marked by dotted arrow in Figure 1). However, the regions differed in the proportion of goblet cell distribution. The cranial region had very few goblet cells as compared to the caudal region. In contrast, goblet cells formed a major fraction of the epithelium in the lung hilar region. Human trachea has comparable number of goblet cells as that of the ferret caudal region but lesser than the ferret lung hilar region (as estimated by visual inspection of H&E stained sections).

### α2–3 glycan receptor distribution in ferret respiratory tract

**Figure 2:**
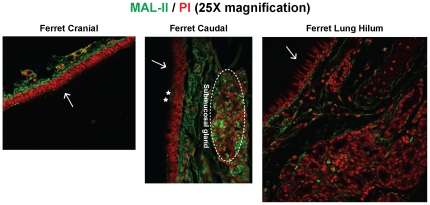
α2–3 linked glycan distribution in ferret respiratory tract. MAL-II lectin (*green*) was used to stain ferret cranial, caudal and lung hilar regions. As seen from the images, MAL-II stained the submucosal glands, the underlying mucosa and some goblet cells (marked as *) in the caudal region. There was no staining of the lung hilar region indicating an absence of α2–3 glycans. The nuclei were stained with PI (*red*). The apical surface is marked with a *white* arrow.

*Maackia amurensis* agglutinin (MAL-II) was used to probe α2→3 sialylated glycan receptor distribution in ferret respiratory tract (**Figure 2**). No staining was observed in the ciliated or non-ciliated cells in the tracheal cranial epithelium. Although, staining of the underlying connective tissue was observed. In the caudal region, there were a very small proportion of goblet cells (∼1%) that showed faint staining with MAL-II, indicating some expression of O-linked α2→3 sialylated glycans. The underlying connective tissue was also extensively stained with MAL-II. In contrast to the ferret trachea, there is lack of expression of α2→3 sialylated glycans in the ferret lung hilar as indicated by a complete lack of staining of both ciliated and non-ciliated cells in the epithelium and in the underlying connective tissue and submucosal glands (**Figure 2**). The minimal to complete lack of expression of α2→3 sialylated glycans in the ferret upper and lower respiratory tract is similar to the minimal expression of these glycan receptors in the human tracheal tissue sections [16], [22].

### O-linked α2–6 glycans in ferret respiratory tract

Matrosovich *et al* [24] have shown that the human influenza A viruses, specifically targeted the non-ciliated goblet cells in the cultures of human airway epithelium. Further when we stained human tracheal tissue section with recombinant hemagglutinins (HAs), we found that HAs from human-adapted influenza A viruses showed intense binding to non-ciliated goblet cells in the apical surface of human tracheal section. Goblet cells are known to express mucin O-linked glycans. Based on this observation, it can be concluded that the HAs of human-adapted influenza A viruses, bind to O-linked α2→6 sialylated glycans expressed on these goblet cells.

**Figure 3:**
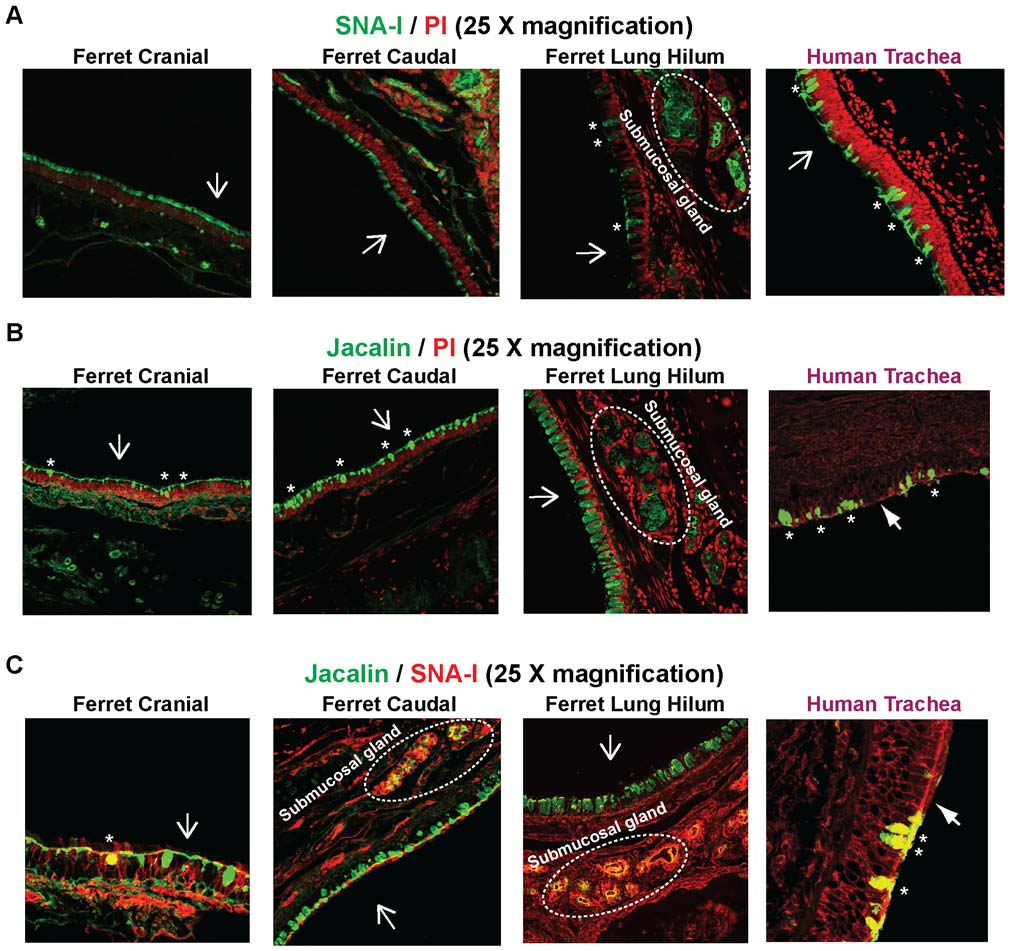
O linked α2–6 glycan distribution in ferret respiratory tract. **A**, Staining of ferret cranial, caudal and lung hilar regions with FITC-labeled SNA-I (*green*). As seen from the images, SNA-I did not stain any of the goblet cells in the ferret trachea (cranial and caudal) although some of the goblet cells (marked as *) in the lung hilar region were stained by SNA-I. Submucosal glands and glycocalyx in both ferret trachea and lung hilar regions showed significant staining with SNA-I. **B**, Staining of ferret cranial, caudal and lung hilar regions with FITC-labeled Jacalin (*green*); a plant lectin having specificity to Tn antigen. Jacalin stained the goblet cells (marked as *), and the submucosal glands in all the three regions of ferret respiratory tract similar to human trachea. **C**, Co-staining of ferret respiratory tissue sections with FITC-labeled Jacalin (*green*) and SNA-I tagged to a 546 nm fluorophore (*red*). Co-staining is seen as *yellow* color due to the overlap of *green* (Jacalin) and *red* (SNA) fluorophores. Submucosal glands in both the trachea and lung hilar region showed significant co-staining with Jacalin/SNA-I. There was some co-staining of goblet cells in the ferret lung hilar region (data not shown) and in ferret trachea cranial region (marked as *). In case of staining with single lectin, the nuclei were visualized by staining with PI (*red*). The apical surface is marked with a *white* arrow.

In order to understand the distribution of O-linked α2→6 glycans in ferret respiratory tract, as a first step, Jacalin was used to probe the distribution of O-linked glycans (**Figure 3B**). All the goblet cells and submucosal glands in the cranial and caudal region of trachea and the lung hilar region that express soluble and membrane-bound mucins showed extensive staining with Jacalin. In the case of human tracheal tissue section, Jacalin staining was predominantly observed in the goblet cell region on the apical surface and not as much in the submucosal glands.

Next, fluorescently labeled SNA-I was used to stain the ferret tissue sections to understand the distribution of α2→6 glycans (**Figure 3A**). The glycocalyx present on the apical side of both cranial and caudal regions and the submucosal gland in ferret trachea showed significant staining with SNA-I. Very few goblet cells in the cranial and caudal regions showed SNA staining, suggesting minimal expression of α2→6 glycan motifs in these cells. In contrast to the ferret tracheal glycan receptor distribution, the ferret lung hilar region showed significant SNA-I staining of goblet cells and submucosal glands although not all of the goblet cells were stained by SNA-I (**Figure 3A**).

In order to probe the distribution of α2→6 sialylated glycans in the context of the abundant O-linked glycans in ferret respiratory tract, a combination of Jacalin and SNA-I was used to co-stain ferret trachea and lung hilar region (co-staining is identified by *yellow* staining pattern in **Figure 3C**). A few goblet cells in the cranial and caudal part of the ferret tracheal section were co-stained by SNA-I and jacalin (co-staining of goblet cells in the cranial part appeared to be more intense than caudal part). There was substantial co-staining of the submucosal glands in the caudal region of the ferret tracheal section. In the case of the ferret lung hilar section, a few goblet cells were co-stained by SNA-I and Jacalin and there was substantial co-staining of the submucosal glands by these lectins. The co-staining pattern of the human tracheal tissue section by SNA-I and Jacalin showed that all the goblet cells on the apical surface were co-stained by these lectins (**Figure 3C**), which was quite different from that observed in the case of the ferret respiratory tract.

### Distribution of their glycan receptors of human-adapted HA in ferret respiratory tract

Representative human-adapted influenza A viruses from the 1918 pandemic (SC18) and from the 1958 pandemic (Alb58) show efficient aerosol transmission in ferrets [5], [23]. The glycan binding properties of the HAs from these viruses have been extensively characterized using glycan array screening analyses [19], [23]. Similar to other human-adapted HAs both SC18 and Alb58 bind with high affinity to glycans having Neu5Acα2→6-linked to longer oligosaccharide motifs (>trisaccharide) such as polylactosamine terminating in α2→6-linked sialic acid (6′SLN-LN) [19], [23]. Although both SC18 and Alb58 show predominant binding to the long α2→6 glycans, they show some differences in their glycan binding specificities. SC18 HA shows highly specific binding primarily to 6′SLN-LN. On the other hand, Alb58 HA has a broader binding specificity to α2→6 sialylated glycans including both short α2→6 oligosaccharide such as 6′SLN and the longer 6′SLN-LN. In addition Alb58 HA also shows observable binding to α2→3 sialylated glycans (3′SLNLN and 3′SLNLNLN) albeit at a much lower affinity than that to α2→6 sialylated glycans [23]. Given that the glycan binding properties of human-adapted HAs have been extensively characterized, recombinant HAs have been used to stain human tracheal and alveolar tissue sections to probe the distribution of glycan receptors for human-adapted influenza A viruses [22], [23], [25]. Using these recombinant HAs to stain the ferret tracheal regions can provide additional details on the distribution of short and long α2→6 sialylated oligosaccharide motifs which are predominantly recognized by human-adapted influenza A viruses.

**Figure 4:**
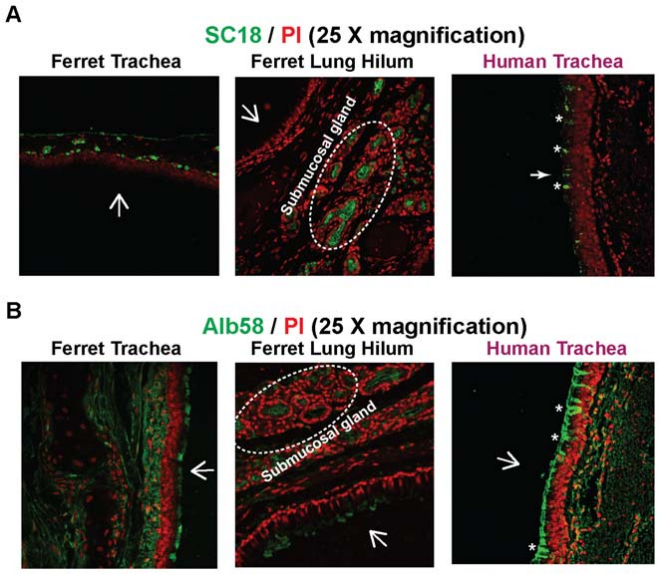
Glycan receptor distribution for human-adapted influenza A virus HA. Recombinant SC18 (H1) HA and Alb58 (H2) HA expressed in insect cells were used to stain ferret cranial, caudal and lung hilar regions at a concentration of 20 µg/ml. **A**, SC18 HA stained only the submucosal glands in all the three regions of ferret respiratory tract. This is in contrast to human trachea where goblet cells (marked as *) were stained by SC18 HA. This restricted binding pattern of SC18 HA can be attributed to its stringent binding specificity to long α2–6 linked (6′SLNLN) glycans. **B**, Alb58 HA stained the submucosal glands, the underlying mucosa and some goblet cells (in the caudal region) similar to SNA-I. This staining pattern is similar to that in human trachea wherein all the goblet cells (marked as *), submucosal glands and the glycocalyx are stained with Alb58HA. The significant goblet cell staining of Alb58 HA of human trachea as compared to ferret respiratory tract is in accordance with predominant expression of O-linked α2–6 sialic acid in human tracheal goblet cells as compared to that in ferret respiratory tract (**Figure 3**). The nuclei were stained with PI (*red*). The apical surface is marked with a *white* arrow.

SC18 HA showed predominant staining of submucosal glands in both ferret tracheal and lung hilar section (**Figure 4A**). It was interesting to note that SC18 HA did not stain goblet cells (which were stained by SNA-I) in lung hilar region. This indicates that α2→6 sialylated glycans comprising of longer oligosaccharide motifs are more extensively distributed in the sub-mucosal glands in comparison with the goblet cells in the hilar region. On the other hand, due to a broader glycan binding specificity of Alb58 HA (binds to both 6′SLN and 6′SLNLN), it stained both the glycocalyx and the underlying submucosal glands in ferret tracheal section (**Figure 4B**). Alb58 HA also stained the goblet cells (similar to SNA-I) and the submucosal glands in the lung hilar section.

**Figure 5:**
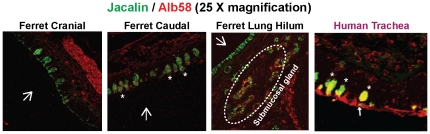
Co-staining of recombinant Alb58 (H2) HA with Jacalin. Ferret cranial, caudal and lung hilar regions were co-stained with 10 µg/ml of Jacalin (FITC labeled) and 20 µg/ml of recombinant Alb58 HA. The co-staining is indicated by a *yellow* staining pattern. As seen from the images, co-staining was predominantly seen in the submucosal glands of ferret trachea and lung hilar region. Some co-staining was also seen in the goblet cells of trachea (marked as *) and lung hilar region. This staining pattern is in contrast to human trachea wherein co-staining is predominantly seen in goblet cells (marked as *). The apical surface is marked with a *white* arrow.

Furthermore, the characteristic pattern of staining by human-adapted HAs such as SC18 and Alb58 is substantial binding to goblet cell regions of human tracheal tissue [19], [23] (**Figure 5**). Hence human-adapted HAs show predominant binding to α2→6 sialylated glycan motifs is seen in the context of O-linked glycans. In the case of ferret respiratory tissues, only faint co-staining of Alb58 HA and Jacalin was observed in a few goblet cells in the apical surface of the cranial and caudal region of tracheal and lung hilar section. This co-staining of goblet cells can be attributed to the presence of some α2,3 sialylated glycans as indicated by staining of some of the goblet cells by MAL-II lectin. On the other hand, Alb58 HA and Jacalin predominantly co-stained the submucosal glands in caudal region of the ferret tracheal (**Figure S1**) and lung hilar sections (**Figure 5**). This pattern indicated a predominant distribution of α2→6 sialylated glycans in the context of O-linked glycans (recognized by human influenza HAs) in submucosal glands of ferret respiratory tract.

In order to verify the sialic acid binding specificity of lectins used in this study, the tissue sections were treated with *Sialidase A* prior to staining with the lectins. *Sialidase A* is an endonuclease that broadly cleaves terminal sialic acids (both α2→3 and α2→6). All the plant lectins and HAs used in this study showed a substantial loss of staining upon *Sialidase A* treatment, thereby confirming that staining of tissues by lectins is due to their specific binding to sialylated glycans on the tissue sections (**Figure S2**).

## Discussion

Ferrets have been used extensively as a model to study influenza A virus transmission and pathogenesis. Upon intranasal inoculation of the virus, ferrets exhibit similar clinical manifestation as that of humans although there seems to be a difference in viral tropism between ferrets and humans. Upper respiratory tract is the primary site of influenza A infection in humans. Apart from upper respiratory tract, involvement of lower respiratory tract (lung hilar region) is also reported in ferrets. Viral tropism is determined by distribution of influenza A glycan receptors, which are recognized by the viral hemagglutinin (HA) during infection.

Plant lectins such as SNA-I have been used to stain ferret tracheal tissues in the past and it is generally known that these tissues predominantly express α2→6 sialylated glycan receptors similar to human tracheal tissues [26]. Although the overall distribution of α2–6 glycans is has been studied in ferret respiratory tract, not much is known about the finer structural details of glycan receptors going beyond the sialic acid linkage. Further in order to understand viral tropism, *in vitro* binding assay to determine the pattern of viral attachment (PVA) of fluorescein labeled human and avian influenza A viral strains to tissues (human and animal) has been performed [3], [27]. Binding of virus was detected by routine immunohistochemical techniques. Although these studies have provided insights into viral binding to specific cell types, they do not fully capture the subtleties of host viral interactions especially in the context of glycan receptor distribution in different cell types [13]. Moreover without the proper understanding of the glycan receptor binding specificites of influenza A viruses (going beyond α2–3/α2–6 linkage specificity) and the glycan receptor distribution in the tissues, it is challenging to extrapolate PVA of a particular viral subtype to its tropism.

**Figure 6:**
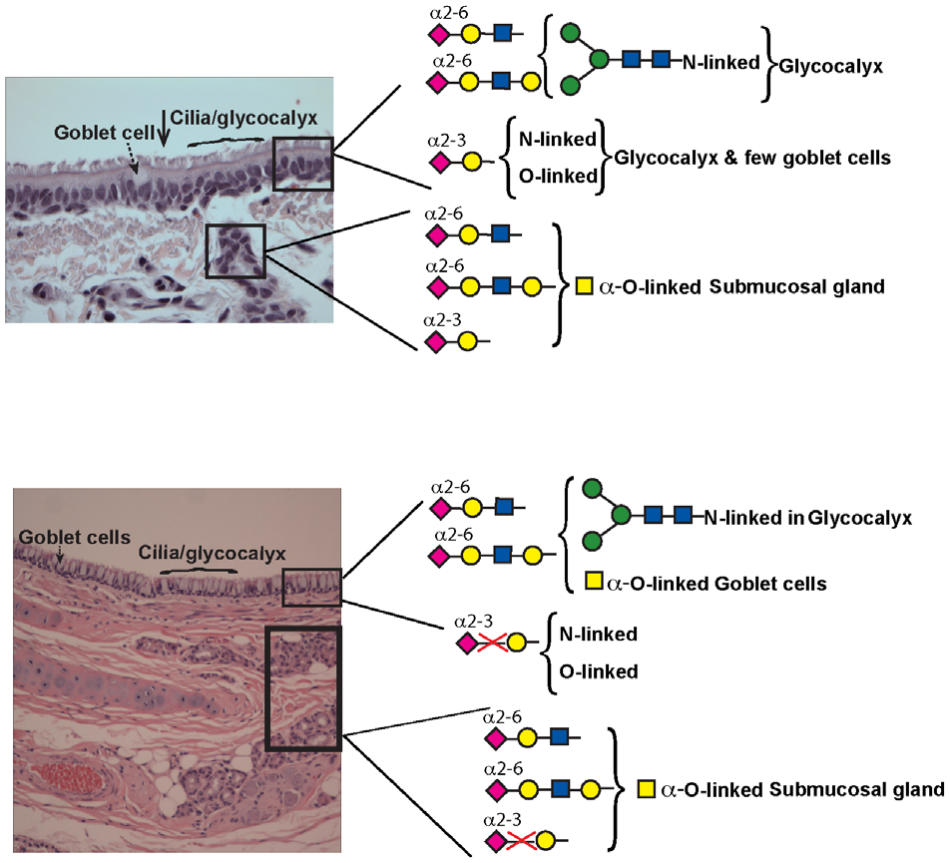
Glycan receptor distribution in ferret respiratory tract. Shown in the figure is the glycan receptor distribution in ferret in ferret trachea (**A**) and lung hilar region (**B**) based on lectin staining patterns. The glycan receptor motifs are shown in cartoon representation (see **Table S1** for cartoon key). Note that predominant α2–6 glycan receptors recognized by human influenza A viruses are found in the submucosal glands of trachea and lung hilum. Further, unlike human respiratory tract, there is minimal to no α2–3 sialylated glycan receptor expression in the lung hilar region of ferret. Except for differences in the number of goblet cells in ferret cranial and caudal region, there were no significant differences in the glycan receptor expression. The apical surface is marked with a *black* arrow.

Recently, with the emergence of glycan array technology, the glycan binding specificities of several plant lectins and influenza A virus hemagglutinins have been extensively characterized. In this study we used a combination of both lectins and recombinant human-adapted HA to systematically stain both the upper and lower respiratory tissues (going all the way to lung hilar region) in ferrets. The use of recombinant human-adapted HAs and co-staining with multiple lectins permitted us to define glycan receptors for human-adapted influenza A viruses going beyond terminal sialic acid linkage and map their distribution parts of the ferret respiratory tract (**Figure 6**).

Our observations support the notion that the receptors for human-adapted influenza A viruses in ferrets are O-linked α2→6 sialylated glycans (based on SNA-I/Jacalin co-staining) that are predominantly distributed in the submucosal glands of the lower respiratory tract (lung hilar region). This notion is also consistent with earlier reports of viral antigens being predominantly found in the submucosal glands in ferret trachea and lung hilar region upon infection with human influenza A virus [28]. This is further corroborated by studies involving the use of recombinant HA to stain human tracheal tissue sections. One of the hallmarks of the human-adapted HA (from influenza A viruses which have caused pandemic and seasonal outbreaks) is their predominant binding to the non-ciliated goblet cells. Even in the cultures of differentiated human airway epithelial cells, the human influenza A viruses are found to predominantly infect non-ciliated cells as compared to avian influenza A viruses which target the ciliated cells [24]. By multiplexing lectin staining with *Sialidase A* treatment, our results suggest that goblet cell region in human tracheal tissue might have a more predominant expression of the sialyl-Tn-antigen motif than the submucosal region in the ferret lung hilum although both these regions appear to be target sites for binding by human-adapted influenza viruses. This result is also supported by recent findings that show increased expression of sialyl-Tn antigen after virus infection due to goblet cell and/or acinar gland neoplasia in ferrets [29].

It is interesting to note that though glycan receptors for human influenza A viruses are differentially distributed in humans and ferrets, they are predominantly expressed in the context of O-linked glycans in either goblet cells (in humans) or in submucosal glands (in ferrets). Given that these O-linked glycans are predominantly expressed in the context of mucins, our study provides a basis to further investigate the role of mucins in influenza A infection. We speculate that by infecting the mucin secreting cells such as goblet cells and submucosal glands, human-adapted influenza A viruses can be easily encapsulated into respiratory droplets formed during sneezing that can in turn facilitate efficient airborne transmission of the virus. In fact, the efficient transmission via respiratory droplets is a hallmark property of human-adapted viruses in the ferret animal models [5].

In summary, using a panel of lectins, we have systematically characterized the glycan receptor distribution for influenza A HA in ferret upper and lower respiratory tract, especially receptors relevant to human adapted viruses (**Figure 6**). This is needed to understand viral pathogenesis in ferrets in order to truly correlate it with that in humans. Although viral pathogenesis is mediated by a concerted function of other viral proteins and host factors, the distribution of host glycan receptors contributes to viral tropism. Moreover, it is important to have a thorough understanding of glycan receptor distribution for improving anti-influenza drug delivery strategies for especially those drugs, which target glycan receptors such as DAS181, a sialidase fusion protein that cleaves off the sialic acid and hence prevents viral entry [30].

## Materials and Methods

### Cloning, baculovirus synthesis, expression and purification of HA

Briefly, recombinant baculoviruses with *WT* HA gene, was used to infect (MOI = 1) suspension cultures of Sf9 cells (Invitrogen, Carlsbad, CA) cultured in BD Baculogold Max-XP SFM (BD Biosciences, San Jose, CA). The infection was monitored and the conditioned media was harvested 3–4 days post-infection. The soluble HA from the harvested conditioned media was purified using Nickel affinity chromatography (HisTrap HP columns, GE Healthcare, Piscataway, NJ). Eluting fractions containing HA were pooled, concentrated and buffer exchanged into 1× PBS pH 8.0 (Gibco) using 100K MWCO spin columns (Millipore, Billerica, MA). The purified protein was quantified using BCA method (Pierce).

### Binding of hemagglutinin to ferret respiratory tissues

Formalin fixed and paraffin embedded ferret tracheal and lung hilar tissue sections were obtained from Lovelace Respiratory Research Institute and Division of Comparative Medicine (Massachusetts Institute of Technology) respectively. The lung hilar tissue sections were obtained from 4 year old male normal ferrets that were not exposed to prior influenza A virus infection. The tracheal sections were also obtained from normal ferrets that were not exposed to prior influenza A virus infection. Tissue sections were deparaffinized, rehydrated and pre-blocked with 1% BSA in PBS for 30 minutes at room temperature (RT). HA-antibody pre-complexes were generated by incubating 20 µg/ml of recombinant HA protein with primary (mouse anti 6× His tag, Abcam) and secondary (Alexa Fluor 488 goat anti mouse IgG, Molecular Probes) antibodies in a ratio of 4∶2∶1 respectively for 20 minutes on ice. Tissue binding studies were performed by incubating tissue sections with the diluted HA-antibody complexes for 3 hours at RT. To visualize the cell nuclei, sections were counterstained with propidium iodide (Invitrogen; 1∶100 in TBST) for 20 minutes at RT. In the case of *Sialidase A* pretreatment, tissue sections were incubated with 0.2 units of *Sialidase A* (recombinant from *Arthrobacter ureafaciens*, Prozyme) for 3 hours at 37°C prior to incubation with the proteins. Sections were then washed and viewed under a Zeiss LSM510 laser scanning confocal microscope.

### Lectin staining of ferret respiratory tract

Formalin fixed and paraffin embedded ferret tracheal and lung hilar tissue sections were obtained from Lovelace Respiratory Research Institute and Division of Comparative Medicine (Massachusetts Institute of Technology) respectively. Tissue sections were deparaffinized and rehydrated. The tissue sections were incubated with 10 µg/ml of lectins (SNA-I, Jacalin and MAL-II) respectively for 3 hours in dark at RT. Lectins were obtained from Vector Labs. To visualize the cell nuclei, sections were counterstained with propidium iodide (Invitrogen; 1∶100 in TBST) for 20 minutes at RT. Sections were then washed, mounted and viewed under a Zeiss LSM510 laser scanning confocal microscope. For MAL-II, since the lectin was biotinylated, prior to adding the lectin, the tissue sections were incubated with streptavidin/biotin kit (Vector Labs) to block endogenous biotin for preventing non-specific staining of the tissues. For lectin co-staining experiments, Jacalin and SNA-I (10 µg/ml each) was added simultaneously to the tissue sections.

## Supporting Information

Table S1 **Glycan binding specificities of lectins used in this study.** Shown in the table is the panel of lectins used in this study and the cartoon representation of glycan motifs recognized by these lectins. The “{“ used to indicate that the glycan motif on the left of “{“ can be linked to either one or more branching positions on the N-linked core glycan structure or different O-linked core glycan structures. Glycan cartoon representation key: *N*-acetyl-D-neuraminic acid (*purple diamond*), D-galactose (*yellow circle*), D-mannose (*green circle*), *N*-acetyl-D-glucosamine (*blue rectangle*), *N*-acetyl-D-galactosamine (*yellow rectangle*).
(PDF)

Figure S1 **Submucosal gland co-staining with Jacalin and Alb58 HA.** Submucosal glands in the ferret trachea showed extensive co-staining with Jacalin and Alb58 HA. The co-staining is indicated by a *yellow* staining pattern. The submucosal glands are marked by *white* dotted circle. The apical surface is marked with a *white* arrow.
(PDF)

Figure S2 **Sialic acid-binding specificity of lectins.** *Sialidase A* from *Arthrobacter ureafaciens* was used to cleave all the sialic acids from the ferret lung hilar tissue sections prior to staining with SNA-I, Alb58 HA and SC18 HA (*green*). A substantial reduction in staining was observed upon *Sialidase A* treatment which indicated sialic acid specific binding of HA and lectins in these tissue sections. The apical surface is marked with a *white* arrow.
(PDF)
