# Supplementary material for: Decoding the Distribution of Glycan Receptors for Human-Adapted Influenza A Viruses in Ferret Respiratory Tract
Source: PLoS One. 2012 Feb 16;7(2):e27517. doi: 10.1371/journal.pone.0027517 (PMC3281014; doi:10.1371/journal.pone.0027517)
Supplement: Table S1 — Glycan binding specificities of lectins used in this study. Shown in the table is the panel of lectins used in this study and the cartoon representation of glycan motifs recognized by these lectins. The “{“ used to indicate that the glycan motif on the left of “{“ can be linked to either one or more branching positions on the N-linked core glycan structure or different O-linked core glycan structures. Glycan cartoon representation key: N-acetyl-D-neuraminic acid (purple diamond), D-galactose (yellow circle), D-mannose (green circle), N-acetyl-D-glucosamine (blue rectangle), N-acetyl-D-galactosamine (yellow rectangle). (PDF) [file pone.0027517.s001.pdf]

Table S1 Glycan binding specificities of lectins used in this study

Lectins	***Glycan Motifs Recognized***
**SNA-I**	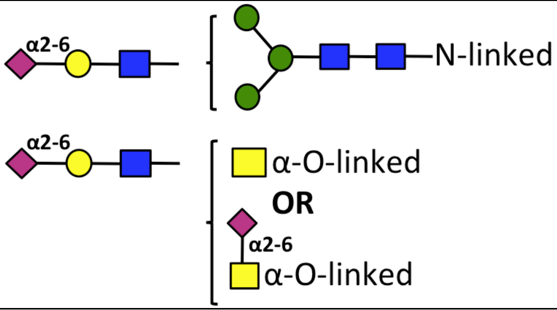
**MAL-II**	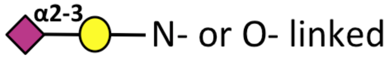
**Jacalin**	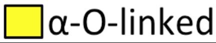
**SNA-I/Jacalin co-staining**	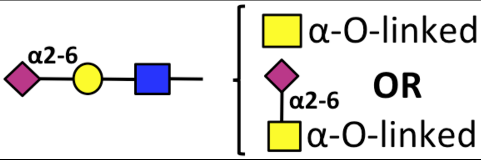
**SC18 HA (1918 H1N1 pandemic)**	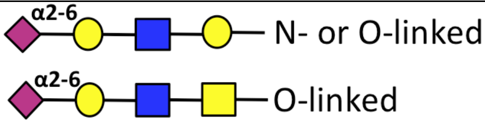
**Alb58 HA (1958 H2N2 pandemic)**	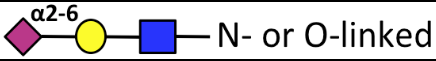
