# Supplementary figures and images for: Decoding the Distribution of Glycan Receptors for Human-Adapted Influenza A Viruses in Ferret Respiratory Tract
Source: PLoS One. 2012 Feb 16;7(2):e27517. doi: 10.1371/journal.pone.0027517 (PMC3281014; doi:10.1371/journal.pone.0027517)

Ferret Caudal Region  
Jacalin/Alb58 (25X magnification)

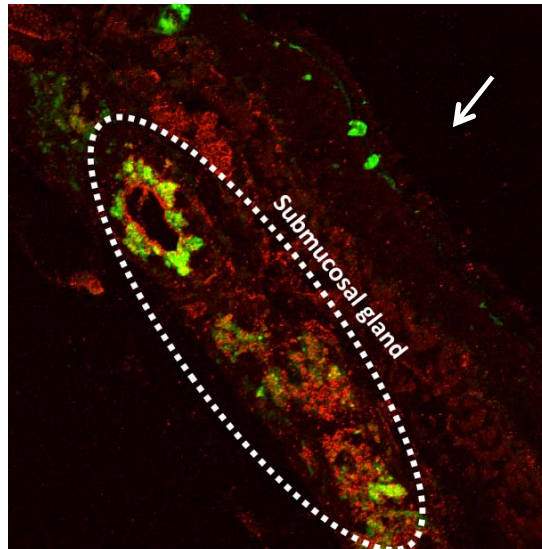

Supplement: Figure S1 — Submucosal gland co-staining with Jacalin and Alb58 HA. Submucosal glands in the ferret trachea showed extensive co-staining with Jacalin and Alb58 HA. The co-staining is indicated by a yellow staining pattern. The submucosal glands are marked by white dotted circle. The apical surface is marked with a white arrow. (PDF) [file pone.0027517.s002.pdf]

A

SNA/PI

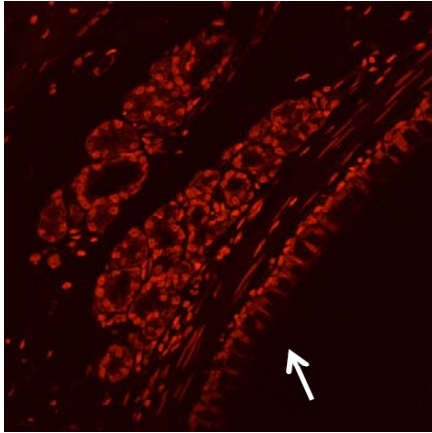

Alb58/PI

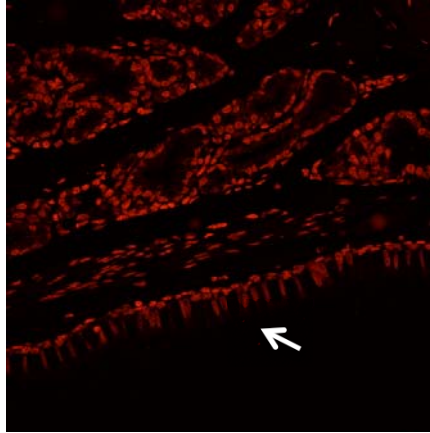

SC18/PI

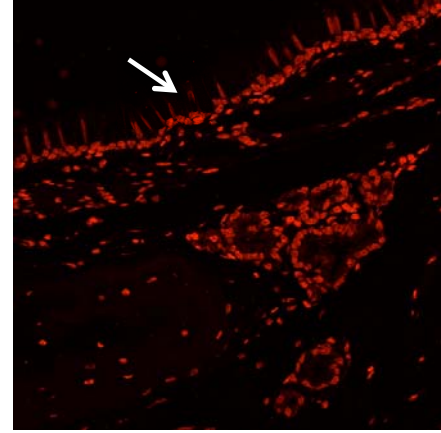

B

SNA/PI + Sialidase A

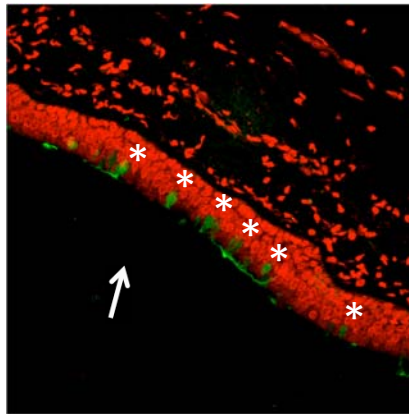

Supplement: Figure S2 — Sialic acid-binding specificity of lectins. Sialidase A from Arthrobacter ureafaciens was used to cleave all the sialic acids from the ferret lung hilar tissue sections prior to staining with SNA-I, Alb58 HA and SC18 HA (green). A substantial reduction in staining was observed upon Sialidase A treatment which indicated sialic acid specific binding of HA and lectins in these tissue sections. The apical surface is marked with a white arrow. (PDF) [file pone.0027517.s003.pdf]
